# Household Tenure and Its Associations with Multiple Long-Term Conditions amongst Working-Age Adults in East London: A Cross-Sectional Analysis Using Linked Primary Care and Local Government Records

**DOI:** 10.3390/ijerph19074155

**Published:** 2022-03-31

**Authors:** Elizabeth Ingram, Manuel Gomes, Sue Hogarth, Helen I. McDonald, David Osborn, Jessica Sheringham

**Affiliations:** 1 Department of Applied Health Research, University College London, London WC1E 7HB, UK; m.gomes@ucl.ac.uk (M.G.); j.sheringham@ucl.ac.uk (J.S.); 2 London Boroughs of Camden and Islington, London N1 1XR, UK; sue.hogarth@islington.gov.uk; 3 London School of Hygiene and Tropical Medicine, London WC1E 7HT, UK; helen.mcdonald@lshtm.ac.uk; 4 Division of Psychiatry, University College London, London W1T 7BN, UK; d.osborn@ucl.ac.uk; 5 Camden and Islington NHS Foundation Trust, London NW1 0PE, UK

**Keywords:** multimorbidity, multiple long-term conditions, comorbidity, social determinants of health, housing, household tenure, data linkage

## Abstract

Multiple long-term conditions (MLTCs) are influenced in extent and nature by social determinants of health. Few studies have explored associations between household tenure and different definitions of MLTCs. This study aimed to examine associations between household tenure and MLTCs amongst working-age adults (16 to 64 years old, inclusive). This cross-sectional study used the 2019–2020 wave of an innovative dataset that links administrative data across health and local government for residents of a deprived borough in East London. Three definitions of MLTCs were operationalised based on a list of 38 conditions. Multilevel logistic regression models were built for each outcome and adjusted for a range of health and sociodemographic factors. Compared to working-age owner-occupiers, odds of basic MLTCs were 36% higher for social housing tenants and 19% lower for private renters (OR 1.36; 95% CI 1.30–1.42; *p* < 0.001 and OR 0.81, 95% CI 0.77–0.84, *p* < 0.001, respectively). Results were consistent across different definitions of MLTCs, although associations were stronger for social housing tenants with physical-mental MLTCs. This study finds strong evidence that household tenure is associated with MLTCs, emphasising the importance of understanding household-level determinants of health. Resources to prevent and tackle MLTCs among working-age adults could be differentially targeted by tenure type.

## 1. Introduction

The co-occurrence of multiple long-term conditions (MLTCs) within a single individual is a major public health challenge both globally and in the UK. The nature and extent of MLTCs is influenced by social determinants of health (SDoH) [1]. The role of individual- and area-level social determinants has been widely reported—prevalence and incidence of MLTCs are greater with increasing age, for women, for ethnic minorities, and those living with greater socioeconomic deprivation [1,2,3,4,5,6]. Yet recent evidence suggests that household-level SDoH (such as household tenure) are often overlooked as determinants of MLTCs despite comparatively large effect sizes for household compared to area-level SDoH [7]. In their landmark report, the Academy of Medical Sciences (AMS) concluded that most evidence focuses on “population or individual-level” determinants and that “it will be valuable to consider whether factors that operate at the household-level can also influence MLTCs” [1]. In addition, exploring these relationships amongst working-age adults has received little attention [1,7,8]. This is despite recent evidence that suggests the median age of onset of MLTCs decreased from 56 years in 2004 to 46 years in 2019 [9].

Household tenure—whether someone privately rents their home, rents from social housing, or owner-occupies—is widely considered a SDoH [10]. In recent years, homeownership in England has increased amongst older adults and decreased in mid-life, with the private rental market increasingly housing working-age adults [11]. First introduced in 1980, the UK Government’s Right to Buy policy and its future iterations enabled some more wealthier social housing tenants to legally buy their properties at a discount, resulting in tenure types more segregated by economic status and social class [12]. Different tenure types are thought to influence health through differences in exposure to various household- and area-level stressors, such as household overcrowding and access to green space [13,14,15]. However, studies examining associations between household tenure and MLTCs report mixed results, have not explored associations in the English context and have not examined interactions between tenure and other household-level sociodemographic circumstances [7]. This is important as evidence suggests that context-specific factors such as degree of homeownership, and supply and conditions of rented housing may profoundly influence the meaning associated with residing in different tenures across geographies and over time [7,14,16,17].

Using an innovative dataset linking data from local government, health and social care, this study aimed to examine and quantify associations between household tenure and MLTCs amongst working-age adults residing in a deprived borough of East London.

## 2. Materials and Methods

### 2.1. Study Design, Data Source and Participants

This cross-sectional study uses the Care City Cohort, which links administrative health and social data across local government services, health providers, and health commissioners for residents of Barking and Dagenham (LBBD) [18]. Data are linked at both individual and household levels. LBBD is a deprived, outer borough of East London, with approximately 211,988 residents and a younger and more ethnically diverse population compared to the rest of England [19]. See Appendix A for an overview of the dataset and data linkage steps. This manuscript was prepared following the RECORD checklist [20].

This study used a cross-section of the primary care and local government data taken on 1st April 2019. Individuals were included if they were of working age (between 16 and 64 years old, inclusive) [21], identified as residents of the borough by Mayhew and Harper’s Residents’ Matrix [22], and were not living in a residential home.

### 2.2. Outcome Measures: MLTCs

MLTCs status was determined based on the presence or absence of 38 long-term conditions recorded in a participant’s primary care record. Flags of these conditions were derived using publicly available code lists [23].

This study operationalised three definitions of MLTCs in consultation with patients and clinicians:

1. Basic MLTCs, the co-occurrence of two or more long-term conditions within a single individual;
2. Physical-mental MLTCs, the co-occurrence of two or more long-term conditions within a single individual, one of which must be depression or anxiety and one of which must be a physical condition;
3. Complex MLTCs, the co-occurrence of three or more long-term conditions affecting three or more different bodily systems within a single individual [24].

The third definition was operationalised as conditions originating from different bodily systems are thought to be harder to treat due to different origins and/or treatment plans [1,24]. See Table A2 for the 38 conditions, how these conditions were grouped by bodily system and their distribution across the study cohort. Binary variables were created to indicate the presence or absence of each MLTCs outcome for each participant.

### 2.3. Main Exposure: Household Tenure

Individuals were defined as “owner-occupiers” if living in an owner-occupied household (outright or with a mortgage), “private renters” if living in a privately rented property, or “social housing tenants” if living in a socially rented household (from local government or a housing association). A fourth “unknown” category was created to account for missing data. Data on tenure were extracted from the council’s housing data systems.

### 2.4. Covariates

Data on age and sex were extracted from primary care records. Eight categories were created to code individuals’ ages in years (<16, 16–29, 30–44, 45–54, 55–64, 65–74, 75–84, and 85+). Sex was coded as male or female. Data on ethnicity were extracted from council records and coded into five categories: “White”, “Black”, “Asian”, “Other” and “Unknown”. Data on BMI and smoking status were extracted from primary care records. BMI was coded into five categories defined by the NHS as follows: underweight (below 18.5), healthy (between 18.5 and 24.9), overweight (between 25 and 29.9), obese (between 30 and 39.9) and morbidly obese (over 40), with a sixth “unknown” category to account for missing data. Smoking status data were coded into four categories: non-smoker, smoker, ex-smoker, or “unknown”.

Data on household welfare benefits, occupancy and household type were extracted from council housing records. Households receiving welfare benefits to support rental payments (‘housing benefit’) were classified by whether eligibility was based on receipt of other welfare benefits and, if so, the type: Employment Support and Allowance (ESA), Pension Credit, Income Support or Job Seeker’s Allowance (JSA). Two further categories reflecting households solely in receipt of housing benefit or in receipt of no benefits were created. Occupancy data were recorded into four categories to reflect 1–2, 3–5, 6–10 and 11 or more people within a household. Data on household type captured households as six types: adults with children, adults with no children, single adult with children, single adult, older adults with no children, and three generations.

To provide a marker of overall deprivation in each participants’ residential area relative to other areas in the borough, borough-specific Index of Multiple Deprivation (IMD) quintiles were calculated for each small geographical area (Lower Super Output Area; LSOA) using 2019 IMD scores [25]. Each LSOA comprised a maximum of 3000 residents and 1200 households [26].

### 2.5. Main Data Analysis

**Figure A1:**
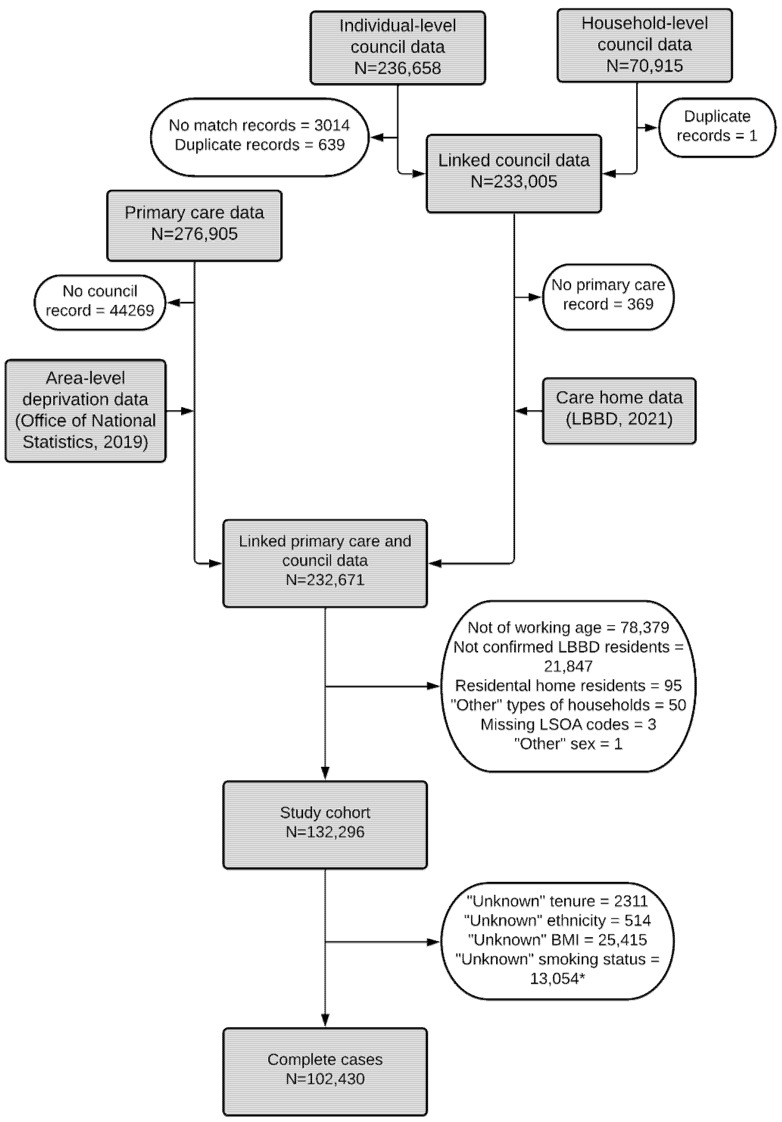
Results of data linkages. * Number of participants with missing data on each variable sum to greater than 29,866 (132,296 minus 102,430) as some participants had missing data across more than one variable.

Multilevel logistic regression modelling was used to explore associations between household tenure and MLTCs prevalence amongst working-age residents with complete data (see Table 1 and Figure A1). To assess the relative impact of adjusting for individual compared to household-level covariates on the association between tenure and MLTCs prevalence, we built three distinct models for each outcome. First, an unadjusted model with no covariates included. Second, a model adjusted for individual-level sociodemographic characteristics available in the dataset and found to be associated with both MLTCs prevalence and household tenure in previous literature [17,27,28,29]. These covariates were age, sex, ethnicity, BMI and smoking. The third and final model for each outcome additionally adjusted for household benefits receipt, occupancy and type to control for potential household-level factors correlated with both household tenure and MLTCs (see covariates above). We chose to adjust for household benefits receipt as it was the best proxy measure available in the dataset for other important covariates such as employment. We chose to adjust for household occupancy and type as a previous systematic review examining household- and area-level social determinants of MLTCs found these factors were associated with MLTCs prevalence in some contexts [7]. Model fit was assessed using Akaike’s Information Criteria (AIC). We considered multilevel models to account for the potential clustering of individuals within geographical areas, as individuals are likely to be more similar in terms of individual, household- and area-level factors if residing in the same areas than if residing in different areas. All models included random effects at the Lower Layer Output Area (LSOA) level to account for clustering within areas. Models were estimated using the lme4 package in R, using restricted maximum likelihood [30]. The 95% confidence intervals were calculated using the Wald test [31].

### 2.6. Subgroup and Sensitivity Analyses

Three interaction terms were separately added to the final model for each outcome to evaluate potential interactions between household tenure and other household factors. We assessed interactions with receipt of benefits, household occupancy and type (see covariates above) as these are most likely to modify the association between housing tenure and MLTCs, and they also act at the household-level. Any differences in these household-level characteristics by tenure type can be found in Table A3.

## 3. Results

### 3.1. Participant Characteristics

Of the 232,671 participants whose primary care and local government records were successfully linked, 132,296 participants were eligible for inclusion in this study. A total of 78,379 records (33.7%) were excluded as individuals were not of working age, 21,847 records (9.39%) were excluded due to unconfirmed resident status and 95 were excluded due to living in a residential home (0.04%) (see Figure A1).

The 132,296 study participants resided in 59,535 households and 110 LSOAs. Table 1 gives an overview of the study participants. A total of 86,770 participants (65.6%) were between the ages of 16 and 44 years old and 68,004 (51.4%) were female. A total of 69,611 (52.6%) were of White ethnicity and 68,631 (51.8%) were overweight, obese or morbidly obese. A total of 54,324 participants (41.1%) were owner-occupiers, 39,885 (30.1%) were private renters and 35,776 (27.0%) were social housing tenants. Crude prevalence of basic, physical-mental, and complex MLTCs was 17.9% (23,683/132,296), 4.7% (6269/132,296) and 6.0% (7931/132,296), respectively.

The number of participants with missing data on tenure, ethnicity, BMI, and smoking status were 2311 (1.75%), 514 (0.39%), 25,415 (19.2%) and 13,054 (9.87%), respectively. A total of 102,430 participants had complete data across all variables and were included in analyses (see Figure A1).

### 3.2. Household Tenure and MLTCs

After adjusting for individual-level characteristics (age, sex, ethnicity, BMI, and smoking), social housing tenants were more likely to have basic MLTCs (OR 1.90; 95% CI 1.83–1.98), physical-mental MLTCs (OR 2.60, 95% CI 2.43–2.79,) and complex MLTCs (OR 2.23, 95% CI 2.10–2.37) when compared to owner-occupiers (Table 2). For private renters, there was no evidence of a difference in the odds of basic MLTCs compared to owner-occupiers (*p* = 0.630). Conversely, private renters were more likely to have physical-mental and complex MLTCs when compared to owner-occupiers (physical-mental MLTCs: OR 1.29, 95% CI 1.19–1.40; complex MLTCs: OR 1.16, 95% CI 1.08–1.25).

After additional adjustment for household-level characteristics (benefits receipt, occupancy, and household type), social housing tenants were still more likely to have MLTCs compared to owner-occupiers, but associations were weaker for all three definitions of MLTCs (basic MLTCs: OR 1.36, 1.30–1.42; physical-mental MLTCs: OR 1.46, 95% CI 1.35–1.58; complex MLTCs: OR 1.34; 95% CI 1.25–1.44). On the other hand, private renters were less likely to have basic MLTCs (OR 0.81, 95% CI 0.77–0.84), physical-mental MLTCs (OR 0.85, 95% CI 0.78–0.93) and complex MLTCs (OR 0.81, 95% CI 0.74–0.87) (Table 2). IMD quintiles were not included in final models for the three MLTCs outcomes as adding these resulted in poorer model fit.

### 3.3. Subgroup Analyses

Our subgroup analyses suggest subgroup effects according to household benefits receipt, occupancy and household type (see Table A4, Table A5 and Table A6). The odds of MLTCs for private renters (compared to owner-occupiers) were considerably stronger for households in receipt of benefits compared to those not receiving benefits. For example, odds of basic MLTCs were 76% greater for privately rented households where someone was in receipt of ESA compared to households not receiving ESA (OR 1.76, 95% CI 1.35–2.29). There was no evidence of an interaction between living in social housing and household benefits receipt (see Table A4). The odds of MLTCs for both social housing tenants and private renters (compared to owner-occupiers) were higher for single-adult households compared to households with adults and children. For example, the odds of basic MLTCs for social housing tenants compared to owner-occupiers were 31% greater for single-adult households (OR 1.31, 95% CI 1.15–1.50). Evidence for subgroup effects for other household types were weaker, with most interactions not statistically significant (see Table A6).

## 4. Discussion

### 4.1. Summary of Study Findings

Risk of MLTCs amongst working-age residents of a deprived East London borough was greater for social housing tenants and lower for privately renters, when compared to owner-occupiers. These associations remained significant after adjusting for a range of individual- and household-level characteristics and were consistent across different definitions of MLTCs. Other household-level variables—household benefits receipt, occupancy, and type—were important modifying factors, with associations between tenure and MLTCs greater for individuals in single-adult households and households in receipt of certain benefits.

### 4.2. Comparisons with Existing Literature

Our prevalence estimates are in keeping with previous estimates for this age group [6,9,32,33]. Prevalence of MLTCs was greater with increasing age and for females, consistent with previous literature [1,6]. However, prevalence was lower for ethnic minority compared with White participants, which contradicts many studies and may be an age-related effect [1,27,32]. In this study, participants lived in a deprived borough in East London where older and younger individuals tend to be White and ethnic minorities, respectively.

We found that social housing tenants exhibited greater risk of MLTCs compared to owner-occupiers, aligning with findings from Northern Ireland yet contradicting those from a Hong Kong-based study [34,35]. This supports the idea that associations between household-level SDoH and MLTCs may be context specific, influenced by housing policy, supply and conditions of social housing, stigma and other household circumstances such as benefits receipt (see Table A3) [7]. In the UK specifically, social housing tenants may be exposed to various “hard” (material) and “soft” (psychological) factors that interact to cause or exacerbate MLTCs [14]. Evidence suggests social housing tenants in the UK have higher levels of C-reactive protein, a biomarker of inflammation associated with various long-term conditions [17,36]. In addition, social housing tenants have less control over the condition of their property and their built environment, and are less able to leave their property, whilst owner-occupying affords ontological security—the sense of security and control afforded when owning your home [37,38]. On top of this, the UK Housing Act (1998) requires social housing to be allocated based on certain criteria, one of which is ill health. As such, MLTCs may be a qualifying characteristic for eligibility for social housing, which may explain our estimated associations.

The lower risk of MLTCs found for private renters compared to owner-occupiers contradicts previous research from the US and Northern Ireland [32,35]. Our analyses adjusted for variables not adjusted for in these studies—household benefits receipt, occupancy, and household type. Our findings suggest these were important explanatory factors for the association between tenure and MLTCs, but they did not explain all of the additional risk experienced by social housing tenants, nor the decreased risk for private renters. In the UK, the private rental market is expanding considerably, and private renters are an increasingly heterogenous group in terms of their demographic, social and economic circumstances [11]. As such, more longitudinal, causal analyses are needed to unpick the complex relationships between different tenure types and MLTCs, taking into account the influence of other household characteristics.

We found that the association between tenure and MLTCs was greater for individuals in single-adult households and households with one or two occupants when compared to higher numbers of occupants. However, previous research examining associations between living alone and MLTC prevalence presents mixed results [7]. In our context, a deprived borough of East London, single-adult households may have less social support and be more financially uncertain than households with multiple occupants, increasing their vulnerability to any adverse effects imposed by their tenure [39]. We also found that the association between tenure and MLTCs was greater for individuals in households where someone was in receipt of certain benefits. Only one previous study has explored subgroup effects in the relationship between tenure and MLTCs and they similarly found that household financial burden mediated this relationship, albeit with a small effect [34]. Our findings support this work, and, again, suggest further research should capture data on, and account for, other household-level characteristics when examining relationships between tenure and MLTCs.

Differences in the risk of MLTCs with tenure type were not explained by commonly used area-level deprivation measures as most areas in our study are amongst the most deprived nationally [7]. These findings further demonstrate the importance of capturing data on, and understanding, household-level SDoH as this information could support service planning when area-level deprivation measures are unable to capture enough variation to model socioeconomic inequalities in MLTCs. In addition, our findings were consistent across different definitions of MLTCs, illustrating the importance of household tenure as a risk factor for MLTCs.

### 4.3. Strengths and Limitations

This is the first study to explore associations between household tenure and MLTCs in England. Our findings add to the current literature, and our analyses would not have been possible without the innovative linkage of primary care and local government data. We operationalised three definitions of MLTCs that captured different types of MLTCs with different degrees of complexity. We used publicly available code lists to determine the presence of each condition.

Our study was conducted in one deprived borough in East London and, whilst our findings could be generalisable to other urban areas, they may not hold in contexts that are less deprived, more rural and have different tenure profiles [7,40]. We restricted our analyses to complete cases, which assumes that any differences between individuals with missing and complete data are explained by differences in observed individual and household characteristics included in the regression models. We recognise that there may be other variables associated with the missing data that we have not adjusted for. However, this is unlikely to have significantly changed the results due to the limited role that BMI and smoking status have in the association between tenure and MLTCs prevalence [41]. We did not account for disease severity or symptom burden on the patient, or other dimensions of MLTCs such as frailty. We may have misclassified households where owner-occupiers privately rented rooms, which may have biased estimates towards the null if private renters who co-resided with their owner-occupying landlords differed systematically in their health compared to private renters who did not. In addition, our measure of household benefits receipt did not capture eligibility for benefits, and we could not adjust for other important factors such as education. The cross-sectional study design did not allow us to explore temporal relationships between tenure and MLTCs. We adjusted for household benefits receipt, occupancy, and household type as potential confounders, but also demonstrated important subgroup effects according to some of these characteristics. It is possible these variables may modify the relationship between tenure and MLTCs. More longitudinal analyses are needed to determine how these factors interact over time to impact MLTCs.

### 4.4. Implications for Practice and Policy

Most interventions for MLTCs focus on retired, older adults, yet our findings indicate that working-age adults are an important population to consider when aiming to address MLTCs. There is currently a gap in models of care or interventions aimed at working-age adults, for whom there may be greater opportunity for prevention of MLTCs through addressing SDoH than amongst older adults [1]. Initiatives that target preventative resources at working-age adults with MLTCs who live in social housing could slow the progression of MLTCs and improve health outcomes, ultimately saving future costs [8].

## 5. Conclusions

This study finds strong evidence that risk of MLTCs amongst working-age residents of a deprived East London borough was greater for social housing tenants and lower for privately renters when compared to owner-occupiers. Associations were consistent across different definitions of MLTCs, which emphasises the importance of understanding and addressing household-level determinants of health. Our findings suggest that resources to prevent and tackle MLTCs could be differentially targeted by tenure type and that working-age adults are an important population to consider in preventative strategies. Further research should employ longitudinal research methods to assess temporal relationships between household social determinants and MLTCs.

## Figures and Tables

**Table 1:** Characteristics of study participants (N = 132,296).

	Total Sample (N = 132,296)	Basic MLTCs (N = 23,683)		Physical-Mental MLTCs (N = 6269)		Complex MLTCs (N = 7931)	
	N (%)	N (%)	Odds Ratio (95% CI)	N (%)	Odds Ratio (95% CI)	N (%)	Odds Ratio (95% CI)
Individual-level variables							
Age							
16–29	37,486 (28.3)	2502 (10.6)	1 (baseline)	578 (9.22)	1 (baseline)	329 (4.15)	1 (baseline)
30–44	49,284 (37.3)	5689 (24.0)	1.82 (1.74–1.92)	1669 (26.6)	2.24 (2.04–2.46)	1274 (16.1)	3.00 (2.66–3.39)
45–54	26,560 (20.1)	7021 (29.6)	5.02 (4.79–5.28)	1898 (30.3)	4.91 (4.47–5.41)	2372 (29.9)	11.1 (9.87–12.5)
55–65	18,966 (14.3)	8471 (35.8)	11.3 (10.7–11.9)	2124 (33.9)	8.05 (7.34–8.85)	3956 (49.9)	29.8 (26.6–33.4)
Sex							
Female	68,004 (51.4)	13,298 (56.1)	1 (baseline)	4112 (65.6)	1 (baseline)	4628 (58.4)	1 (baseline)
Male	64,292 (48.6)	10,385 (43.9)	0.79 (0.77–0.82)	2157 (34.4)	0.54 (0.51–0.57)	3303 (41.6)	0.74 (0.71–0.78)
Ethnicity							
White	69,611 (52.6)	14,472 (61.1)	1 (baseline)	4755 (75.8)	1 (baseline)	5181 (65.3)	1 (baseline)
Black	28,335 (21.4)	4178 (17.6)	0.66 (0.63–0.68)	617 (9.84)	0.30 (0.28–0.33)	1174 (14.8)	0.54 (0.50–0.57)
Asian	31,879 (24.1)	4822 (20.4)	0.68 (0.66–0.70)	852 (13.6)	0.37 (0.35–0.40)	1524 (19.2)	0.62 (0.59–0.66)
Other	1957 (1.48)	175 (0.74)	0.37 (0.32–0.44)	40 (0.64)	0.28 (0.20–0.38)	40 (0.50)	0.26 (0.19–0.35)
Unknown	514 (0.39)	36 (0.15)	0.29 (0.20–0.40)	5 (0.08)	0.13 (0.05–0.29)	12 (0.15)	0.30 (0.16–0.50)
BMI categories							
Underweight	4332 (3.27)	522 (2.20)	0.85 (0.77–0.94)	144 (2.30)	0.89 (0.74–1.05)	119 (1.50)	0.72 (0.60–0.87)
Healthy weight	33,918 (25.6)	4707 (19.9)	1 (baseline)	1264 (20.2)	1 (baseline)	1274 (16.1)	1 (baseline)
Overweight	35,870 (27.1)	6854 (28.9)	1.47 (1.41–1.53)	1677 (26.8)	1.27 (1.18–1.37)	2143 (27.0)	1.63 (1.52–1.75)
Obese	27,941 (21.1)	7893 (33.3)	2.44 (2.35–2.54)	2142 (34.2)	2.14 (2.00–2.30)	3045 (38.4)	3.13 (2.93–3.35)
Morbidly obese	4820 (3.64)	2106 (8.90)	4.82 (4.51–5.14)	709 (11.3)	4.46 (4.04–4.91)	1001 (12.6)	6.72 (6.14–7.34)
Unknown	25,415 (19.2)	1601 (6.76)	0.42 (0.39–0.44)	333 (5.31)	0.34 (0.30–0.39)	349 (4.40)	0.36 (0.32–0.40)
Smoking status							
Non-smoker	77,913 (58.9)	12,972 (54.8)	1 (baseline)	2851 (45.5)	1 (baseline)	4117 (51.9)	1 (baseline)
Ex-smoker	15,834 (12.0)	4536 (19.2)	2.01 (1.93–2.09)	1366 (21.8)	2.49 (2.32–2.66)	1806 (22.8)	2.32 (2.18–2.45)
Smoker	25,495 (19.3)	5849 (24.7)	1.49 (1.44–1.54)	2013 (32.1)	2.26 (2.13–2.39)	1987 (25.1)	1.52 (1.43–1.60)
Unknown	13,054 (9.87)	326 (1.38)	0.13 (0.11–0.14)	39 (0.62)	0.08 (0.06–0.11)	21 (0.26)	0.03 (0.02–0.04)
Household-level variables							
Tenure							
Owner-occupied	54,324 (41.1)	9278 (39.2)	1 (baseline)	1801 (28.7)	1 (baseline)	2853 (36.0)	1 (baseline)
Privately rented	39,885 (30.1)	5143 (21.7)	0.72 (0.69–0.75)	1328 (21.2)	1.00 (0.93–1.08)	1554 (19.6)	0.73 (0.69–0.78)
Social housing	35,776 (27.0)	9004 (38.0)	1.63 (1.58–1.69)	3085 (49.3)	2.75 (2.59–2.92)	3450 (43.5)	1.93 (1.83–2.03)
Unknown	2311 (1.75)	258 (1.09)	0.61 (0.53–0.69)	55 (0.88)	0.71 (0.54–0.92)	74 (0.93)	0.60 (0.47–0.75)
Benefit receipt							
None	116,306 (87.9)	18,615 (78.6)	1 (baseline)	4184 (66.7)	1 (baseline)	4569 (57.6)	1 (baseline)
ESA	5636 (4.26)	2926 (12.4)	6.02 (5.69–6.36)	1499 (23.9)	11.3 (10.5–12.1)	1652 (20.8)	9.09 (8.52–9.69)
Pension	1842 (1.39)	489 (2.06)	2.01 (1.81–2.23)	130 (2.07)	2.36 (1.96–2.82)	183 (2.31)	2.42 (2.06–2.82)
Income support	2491 (1.88)	758 (3.20)	2.44 (2.23–2.66)	243 (3.88)	3.36 (2.92–3.84)	300 (3.78)	3.00 (2.65–3.39)
JSA	638 (0.48)	168 (0.71)	1.99 (1.66–2.37)	597 (0.65)	2.13 (1.53–2.90)	63 (0.79)	2.40 (1.83–3.09)
Housing benefit only	5383 (4.07)	727 (3.07)	1.40 (1.34–1.46)	172 (2.74)	2.14 (1.99–2.29)	1164 (14.7)	1.61 (1.51–1.72)
Occupancy (number of people in household)							
1–2	29,082 (22.0)	7878 (33.3)	1 (baseline)	2557 (40.8)	1 (baseline)	3117 (39.3)	1 (baseline)
3–5	76,291 (57.7)	12,438 (52.5)	0.52 (0.51–0.54)	3040 (48.5)	0.43 (0.41–0.45)	3855 (48.6)	0.44 (0.42–0.47)
6–10	25,251 (19.1)	3205 (13.5)	0.39 (0.37–0.41)	639 (10.2)	0.27 (0.25–0.29)	913 (11.5)	0.31 (0.29–0.34)
11+	1672 (1.26)	162 (0.68)	0.29 (0.24–0.34)	33 (0.53)	0.21 (0.14–0.29)	46 (0.58)	0.24 (0.17–0.31)
Household type							
Adults with children	60,648 (45.8)	7715 (32.6)	1 (baseline)	1767 (28.2)	1 (baseline)	2110 (26.6)	1 (baseline)
Adults with no children	41,974 (31.7)	9023 (38.1)	1.88 (1.82–1.94)	2344 (37.4)	1.97 (1.85–2.10)	3269 (41.2)	2.34 (2.22–2.48)
Single adult with children	7274 (5.50)	1103 (4.66)	1.23 (1.14–1.31)	355 (5.66)	1.71 (1.52–1.92)	254 (3.20)	1.00 (0.88–1.14)
Single adult	10,675 (8.07)	3341 (14.1)	3.13 (2.98–3.28)	1212 (19.3)	4.27 (3.95–4.61)	1405 (17.7)	4.20 (3.92–4.51)
Older cohabiting adults	7226 (5.46)	1865 (7.87)	2.39 (2.25–2.53)	467 (7.45)	2.30 (2.07–2.55)	695 (8.76)	2.95 (2.70–3.23)
Three generations	4499 (3.40)	636 (2.69)	1.13 (1.03–1.23)	124 (1.98)	0.94 (0.78–1.13)	198 (2.50)	1.28 (1.10–1.48)
Area-level variables							
Index of Multiple Deprivation Quintiles *							
1 (least deprived)	27,305 (20.6)	4538 (19.2)	1 (baseline)	1017 (16.2)	1 (baseline)	1467 (18.5)	1 (baseline)
2	26,338 (19.9)	4401 (18.6)	1.01 (0.96–1.05)	1068 (17.0)	1.09 (1.00–1.19)	1374 (17.3)	0.97 (0.90–1.05)
3	26,556 (20.1)	4718 (19.9)	1.08 (1.04–1.13)	1271 (20.3)	1.30 (1.19–1.41)	1589 (20.0)	1.12 (1.04–1.21)
4	25,868 (19.6)	4778 (20.2)	1.14 (1.09–1.19)	1328 (19.5)	1.40 (1.29–1.52)	1617 (20.4)	1.17 (1.09–1.26)
5 (most deprived)	26,229 (19.8)	5248 (22.2)	1.25 (1.20–1.31)	1585 (19.6)	1.66 (1.53–1.80)	1884 (23.8)	1.36 (1.27–1.46)

Note: the denominator for all characteristics (individual and household) is the number of individuals. OR = odds ratio; 95% CI = 95% confidence interval; ESA = Employment Support and Allowance, and JSA = Job Seeker’s Allowance. * Calculated for Barking and Dagenham based on raw Index of Multiple Deprivation scores (2019) [25].

**Table 2:** Estimated odds ratios of multiple long-term conditions (MLTCs) with household tenure for working-age adult residents with complete data (N = 102,430).

	MLTCs Prevalence	Model 1 ^a^ Unadjusted OR (95% CI)	*p* Value	Model 2 ^b^ Adjusted OR (95% CI)	*p* Value	Model 3 ^c^ Adjusted OR (95% CI)	*p* Value
	N (%)						
Basic MLTCs							
Household tenure							
OOC * (ref)	8645 (39.8)	-		-		-	
Social housing	8269 (38.1)	1.82 (1.76–1.89)	<0.001	1.90 (1.83–1.98)	<0.001	1.36 (1.30–1.42)	<0.001
Privately rented	4805 (22.1)	0.76 (0.73–0.79)	<0.001	1.01 (0.97–1.05)	0.630	0.81 (0.77–0.84)	<0.001
Variance Partition Coefficient (%)		2.96		1.42		1.14	
Physical-mental MLTCs							
Household tenure							
OOC * (ref)	1705 (29.1)	-		-		-	
Social housing	2894 (49.4)	3.03 (2.83–3.23)	<0.001	2.60 (2.43–2.79)	<0.001	1.46 (1.35–1.58)	<0.001
Privately rented	1261 (21.5)	1.08 (1.00–1.17)	0.042	1.29 (1.19–1.40)	<0.001	0.85 (0.78–0.93)	<0.001
Variance Partition Coefficient (%)		6.05		1.98		1.31	
Complex MLTCs							
Household tenure							
OOC * (ref)	2740 (36.6)	-		-		-	
Social housing	3276 (43.7)	2.12 (2.00–2.25)	<0.001	2.23 (2.10–2.37)	<0.001	1.34 (1.25–1.44)	<0.001
Privately rented	1479 (19.7)	0.77 (0.72–0.83)	<0.001	1.16 (1.08–1.25)	<0.001	0.81 (0.74–0.87)	<0.001
Variance Partition Coefficient (%)		4.11		1.36		0.88	

Complex MLTCs = the co-occurrence of three or more long-term conditions affecting three or more different body systems within a single individual. * OOC = owner-occupied. ^a^ Model 1—an unadjusted model with no covariates. ^b^ Model 2—model adjusted for individual-level covariates: age, sex, ethnicity, BMI and smoking status. ^c^ Model 3—model adjusted for model 2 covariates plus household benefits receipt, household occupancy and household type.

## Data Availability

Restrictions apply to the availability of these data. Data were obtained from Care City and no applicable data are available without their permission. The study protocol is available on request.

## Appendix A. Overview of the Care City Cohort and Data Linkage Steps

In 2017, the leaders of Barking and Dagenham Council, North East London NHS Foundation Trust (NELFT) and Barking and Dagenham, Havering and Redbridge Clinical Commissioning Group (BHR CCG), and their Caldicott guardians (a senior person within each organisation who is responsible for protecting the confidentiality of people’s health and care information and making sure it is used properly), signed data sharing agreements to create a dataset that linked administrative data for the population of Barking and Dagenham (B&D) between 1st April 2011 and 31st March 2017. Since its creation, the dataset has been updated on an annual basis. It is hosted in the Barking and Dagenham, Havering, and Redbridge NHS Accredited Data Safe Haven, with governance and oversight provided by the Barking and Dagenham, Havering, and Redbridge Information Governance Steering Committee.

The dataset was created as part a larger research programme of work [18]. It contains routinely collected administrative health and social data across local government services, health providers, and health commissioners. Data are linked at the individual and household levels using linkage keys (replacing NHS numbers and Unique Property Reference Numbers; UPRNs). The data are pseudonymised and include information on sociodemographic characteristics, health variables, household variables and data on health and social care service utilisation. Data on all sociodemographic and health variables for each cross-section are taken as a snapshot on 1st April 2019 to account for in-year changes in variables. The dataset is not currently publicly available but was made available to the wider research community in Autumn 2020.

More information on the dataset can be found here [42] and here [43]. More information on the codes and algorithms used to classify variables as part of the creation of the Care City Cohort can be found at this reference [18].

This study used data from the 2019/20 cross-section of the Care City Cohort. We requested access to pseudonymised sociodemographic and health variables extracted from primary care data, and resident data extracted from local government data. We did not have access to other data available within the Care City Cohort, such as data on health and care service utilisation.

Data were provided unlinked with linkage keys, i.e., with the identification codes generated to replace NHS numbers and UPRNs. We used these to link the data at the individual and household levels. First, we linked the individual- and household-level local government data on Household_ID (the household-level identification code created by Care City to replace UPRNs). Second, we linked the individual-level primary care data to the linked local government data on Patient_ID (the individual-level identification code created by Care City to replace NHS numbers). Third, we linked a fourth dataset provided by Care City that detailed care homes in Barking and Dagenham and their Household_IDs. We linked this to the cohort data on Household_ID. Finally, we linked a fifth dataset from ONS that contained area-level deprivation data from 2019. We linked this dataset to the data on LSOA code (a unique number identifying each small area/LSOA in England). All linkages were conducted in R software using the merge function from the R base package. Figure A1 illustrates the results of the linkages of the separate primary care and local government datasets. A total of 232,671 individuals were linked across primary care and local government datasets (84.0% of the original primary care records).

To assess whether there were any potential selection biases in the linkage results, we calculated standardised differences in key variables for matched and unmatched primary care records [44]. Standardised differences of 0.2, 0.5, and 0.8 indicate small, medium and large effect sizes, respectively [44]. We were not able to assess potential biases in social variables extracted from local government records (i.e., in the household tenure variable and other household variables) as, by definition, unmatched primary care records did not have corresponding local government data. However, the number of unmatched local government records was considerably low (N = 369). Table A1 presents the results of analyses conducted to assess potential biases in the linkage results for matched and unmatched primary care records. These results indicate that selection biases were not introduced in selected variables originating from primary care records as a result of the success of data linkages, which is in keeping with previous analyses of this data [18].

**Table A1:** Results of analyses to assess potential biases in the linkage results for matched (N = 232,671) and unmatched (N = 44,269) primary care records.

	Primary Care Matched Records N = 232,671	Primary Care Unmatched Records N = 44,269	Standardised Difference
Age: N (%)			
<16	57,402 (24.7)	8877 (20.1)	0.150
16–29	42,325 (18.2)	8593 (19.4)	
30–44	59,891 (25.7)	11,942 (27.0)	
45–54	30,738 (13.2)	5679 (12.8)	
55–64	21,338 (9.17)	4101 (9.26)	
65–74	11,602 (4.99)	2461 (5.56)	
75–84	6366 (2.74)	1414 (3.19)	
85+	3009 (1.29)	1202 (2.72)	
Sex: N (%)			
Female	116,186 (49.9)	21,787 (49.2)	0.025
Male	116,484 (50.1)	22,472 (50.8)	
Other/Missing	1 (0.00)	10 (0.00)	
Ethnicity *: N (%)			
White	76,524 (32.9)	13,633 (31.4)	0.128
Black	32,708 (14.1)	5029 (11.4)	
Asian	42,222 (18.1)	9710 (21.9)	
Mixed	6285 (2.70)	1137 (2.57)	
Other	4309 (1.85)	831 (1.88)	
Unknown	67,493 (29.0)	13,629 (30.8)	
Basic MLTCs: N (%)			
Present	41,329 (17.8)	7931 (17.9)	0.004
Absent	191,342 (82.2)	36,338 (82.1)	
Physical-mental MLTCs: N (%)			
Present	9077 (3.90)	1542 (3.48)	0.022
Absent	223,594 (96.1)	42,727 (96.5)	
Complex MLTCs N (%):			
Present	17,721 (7.65)	3562 (8.09)	0.016
Absent	214,950 (92.4)	40,707 (91.6)	
BMI categories: N (%)			
Underweight	11,645 (5.00)	2115 (4.78)	0.077
Healthy weight	48,101 (20.7)	10,355 (23.4)	
Overweight	49,180 (21.1)	9493 (21.4)	
Obese	37,566 (16.1)	6612 (14.9)	
Morbidly obese	6077 (2.61)	934 (2.11)	
Unknown	80,102 (34.4)	14,760 (33.3)	
Smoking status: N (%)			
Non-smoker	107,326 (46.1)	21,247 (48.0)	0.043
Ex-smoker	24,385 (10.5)	4620 (10.4)	
Smoker	33,722 (14.5)	6372 (14.4)	
Unknown	67,238 (28.9)	12,030 (27.2)	

MLTCs = multiple long-term conditions. * Variable taken from primary care records, unlike in the study analyses.

**Table A2:** The 38 long-term conditions grouped by 10 bodily systems and their distribution across the study cohort (N = 132,296).

**Respiratory**	**N (%)**
Asthma (currently treated)	6551 (4.95)
Bronchiectasis	143 (0.11)
Chronic obstructive pulmonary disorder	1325 (1.00)
Sensory	
Blindness and low vision	905 (0.68)
Chronic sinusitis	1617 (1.22)
Hearing loss	4726 (3.57)
Psoriasis or eczema	812 (0.61)
Cardiovascular	
Atrial fibrillation	451 (0.34)
Coronary heart disease	1446 (1.09)
Heart failure	302 (0.23)
Hypertension	14,518 (11.0)
Peripheral vascular disease	169 (0.13)
Endocrine	
Diabetes	8728 (6.60)
Thyroid disorders	4403 (3.33)
Cancer	
Cancer (in last 5 years)	1157 (0.87)
Musculoskeletal	
Painful conditions	7417 (5.61)
Rheumatoid arthritis (or other inflammatory polyarthropathies and systematic connective tissue disorders)	2871 (2.17)
Mental health	
Alcohol problems	1170 (0.88)
Anorexia and bulimia	820 (0.62)
Anxiety (and other neurotic, stress-related and somatoform disorders)	3935 (2.97)
Depression	9055 (6.84)
Dementia	58 (0.04)
Psychoactive substance misuse	1451 (1.10)
Schizophrenia and bipolar	8624 (6.52)
Neurological	
Epilepsy (currently treated)	750 (0.57)
Learning disability	905 (0.68)
Migraine	331 (0.25)
Stroke and transient ischaemic attack	844 (0.64)
Multiple sclerosis	177 (0.13)
Parkinson’s disease	54 (0.04)
Genitourinary	
Chronic kidney disease	444 (0.34)
Prostate disorders	666 (0.50)
Gastrointestinal	
Chronic liver disease and viral hepatitis	1341 (1.01)
Constipation (treated)	741 (0.56)
Diverticular disease of intestine	893 (0.68)
Irritable bowel syndrome	3914 (2.96)
Inflammatory bowel disease	718 (0.54)
Peptic ulcer disease	760 (0.57)

**Table A3:** Household benefits receipt, occupancy, and household type, by tenure for complete cases (N = 102,430).

	Owner-Occupied N = 43,444	Social Housing N = 27,766	Privately Rented N = 31,220
Household benefits receipt: N (%)			
None	41,670 (95.9)	18,140 (65.3)	21,337 (68.3)
ESA	374 (0.86)	2883 (10.4)	1294 (4.14)
Pension	405 (0.93)	676 (2.43)	419 (1.34)
Income Support	135 (0.31)	1143 (4.12)	612 (1.96)
JSA	32 (0.07)	309 (1.11)	134 (0.43)
Housing benefit only	828 (1.91)	4615 (16.6)	7424 (23.8)
Household occupancy: N (%)			
1–2	9044 (20.8)	8668 (31.2)	6423 (20.6)
3–5	26,458 (60.9)	15,316 (55.2)	16,978 (54.4)
6–10	7531 (17.3)	3668 (13.2)	7153 (22.9)
11+	411 (0.95)	114 (0.41)	666 (2.13)
Household type: N (%)			
Adults with children	17,749 (40.9)	10,884 (39.2)	17,313 (55.5)
Adults with no children	15,888 (36.6)	8917 (32.1)	7164 (22.9)
Single adult with children	1179 (2.71)	2176 (7.84)	2827 (9.06)
Single adult	2948 (6.79)	3593 (12.9)	2380 (7.62)
Older cohabiting adults	3615 (8.32)	1602 (5.77)	660 (2.11)
Three generations	2065 (4.75)	594 (2.14)	876 (2.81)

Note: the denominator for all variables is the number of individuals rather than households. +ESA = Employment Support and Allowance; JSA = Job Seeker’s Allowance.

**Table A4:** Estimated odds ratios of basic, physical-mental, and complex MLTCs with household tenure when the final models tested for interactions between tenure and household benefits receipt for working-age adults residing in B&D in 2019/20 (N = 102,430).

Independent Variables		Basic MLTCs		Physical-Mental MLTCs		Complex MLTCs	
		OR (95% CI)	*p* Value	OR (95% CI)	*p* Value	OR (95% CI)	*p* Value
Tenure	OOC	-	-	-	-	-	-
	Privately rented	0.78 (0.74–0.82)	<0.001	0.77 (0.69–0.86)	<0.001	0.77 (0.70–0.84)	<0.001
	Social housing	1.38 (1.32–1.45)	<0.001	1.54 (1.41–1.68)	<0.001	1.34 (1.24–1.45)	<0.001
Household benefits receipt	No benefits	-	-	-	-	-	-
	ESA	4.21 (3.35–5.28)	<0.001	7.83 (6.04–10.1)	<0.001	6.85 (5.33–8.79)	<0.001
	Pension credit	1.52 (1.19–1.94)	<0.001	1.67 (1.08–2.57)	0.021	1.62 (1.14–2.31)	0.008
	Income support	2.78 (1.90–4.06)	<0.001	2.29 (1.24–4.25)	0.008	2.39 (1.44–3.97)	<0.001
	JSA	0.96 (0.41–2.24)	0.924	0.72 (0.10–5.39)	0.752	1.60 (0.53–4.79)	0.401
	Housing benefit only	1.92 (1.63–2.26)	<0.001	2.45 (1.89–3.17)	<0.001	1.92 (1.52–2.43)	<0.001
Tenure*Household benefitsreceipt	Privately rented*no benefits	-	-	-	-	-	-
	Privately rented*ESA	1.76 (1.35–2.29)	<0.001	1.42 (1.05–1.93)	0.024	1.36 (1.01–1.83)	0.043
	Privately rented*pension credit	1.40 (1.00–1.96)	0.052	1.86 (1.05–3.31)	0.034	1.57 (0.96–2.58)	0.073
	Privately rented*income support	1.08 (0.71–1.66)	0.711	1.47 (0.73–2.93)	0.279	1.57 (0.87–2.84)	0.137
	Privately rented*JSA	2.31 (0.90–5.90)	0.080	1.97 (0.22–17.4)	0.541	1.53 (0.44–5.36)	0.509
	Privately rented*housing benefit only	0.92 (0.78–1.10)	0.356	0.95 (0.71–1.28)	0.752	1.03 (0.79–1.36)	0.807
Tenure*Household benefits receipt (continued)	Social housing*no benefits	-	-	-	-	-	-
	Social housing*ESA	1.12 (0.88–1.43)	0.359	0.74 (0.56–0.99)	0.039	0.95 (0.72–1.25)	0.708
	Social housing*pension credit	1.01 (0.75–1.36)	0.956	0.93 (0.57–1.54)	0.784	0.93 (0.60–1.42)	0.724
	Social housing*income support	0.93 (0.62–1.39)	0.714	1.14 (0.60–2.18)	0.683	1.56 (0.91–2.68)	0.106
	Social housing*JSA	1.59 (0.66–3.86)	0.303	1.96 (0.25–15.1)	0.519	1.08 (0.34–3.42)	0.895
	Social housing*housing benefit only	0.96 (0.80–1.15)	0.681	1.01 (0.76–1.33)	0.966	1.21 (0.94–1.57)	0.142

**Table A5:** Estimated odds ratios of basic, physical-mental, and complex MLTCs with household tenure when the final models tested for interactions between tenure and household occupancy for working-age adults residing in B&D in 2019/20 (N = 102,430).

Independent Variables		Basic MLTCs		Physical-Mental MLTCs		Complex MLTCs	
		OR (95% CI)	*p* Value	OR (95% CI)	*p* Value	OR (95% CI)	*p* Value
Tenure	OOC	-	-	-	-	-	-
	Privately rented	0.99 (0.91–1.08)	0.800	1.04 (0.90–1.20)	0.564	1.02 (0.89–1.16)	0.782
	Social housing	1.54 (1.43–1.66)	<0.001	1.57 (1.40–1.77)	<0.001	1.61 (1.45–1.79)	<0.001
Occupancy categories	1–2 occupants	-	-	-	-	-	-
	3–5 occupants	1.03 (0.96–1.10)	0.415	0.98 (0.87–1.11)	0.781	1.04 (0.94–1.15)	0.464
	6–10 occupants	1.02 (0.93–1.13)	0.611	0.82 (0.67–0.99)	0.041	1.12 (0.96–1.31)	0.141
	11+ occupants	0.95 (0.70–1.28)	0.727	0.68 (0.32–1.47)	0.326	1.29 (0.80–2.08)	0.291
Tenure*Occupancy	Privately rented*1–2 occupants	-	-	-	-	-	-
	Privately rented*3–5 occupants	0.77 (0.70–0.85)	<0.001	0.73 (0.61–0.87)	<0.001	0.74 (0.63–0.87)	<0.001
	Privately rented*6–10 occupants	0.73 (0.64–0.83)	<0.001	0.80 (0.62–1.03)	0.089	0.66 (0.53–0.81)	<0.001
	Privately rented*11+ occupants	0.69 (0.46–1.03)	0.072	0.88 (0.35–2.21)	0.791	0.37 (0.18–0.75)	0.006
	Social housing*1–2 occupants	-	-	-	-	-	-
	Social housing*3–5 occupants	0.84 (0.77–0.92)	<0.001	0.88 (0.77–1.02)	0.093	0.78 (0.69–0.89)	<0.001
	Social housing*6–10 occupants	0.86 (0.75–0.97)	0.019	0.97 (0.76–1.23)	0.794	0.63 (0.51–0.78)	<0.001
	Social housing*11+ occupants	0.42 (0.22–0.78)	0.007	0.86 (0.29–2.55)	0.785	0.42 (0.17–1.04)	0.060

**Table A6:** Estimated odds ratios of basic, physical-mental, and complex MLTCs with household tenure when the final models tested for interactions between tenure and household type for working-age adults residing in B&D in 2019/20 (N = 102,430).

Independent Variables		Basic MLTCs		Physical-Mental MLTCs		Complex MLTCs	
		OR (95% CI)	*p* Value	OR (95% CI)	*p* Value	OR (95% CI)	*p* Value
Tenure	OOC	-		-	-	-	-
	Privately rented	0.75 (0.70–0.81)	<0.001	0.81 (0.70–0.92)	<0.001	0.67 (0.59–0.75)	<0.001
	Social housing	1.40 (1.30–1.50)	<0.001	1.54 (1.35–1.75)	<0.001	1.22 (1.09–1.38)	<0.001
Household type	Adults with children	-	-	-	-	-	-
	Adults with no children	1.27 (1.19–1.35)	<0.001	1.28 (1.13–1.46)	<0.001	1.17 (1.05–1.30)	0.003
	Single adult with children	0.92 (0.76–1.11)	0.364	0.78 (0.53–1.17)	0.233	0.72 (0.49–1.07)	0.103
	Single adult	1.16 (1.04–1.30)	0.009	1.46 (1.20–1.78)	<0.001	1.01 (0.85–1.21)	0.892
	Older cohabiting adults	1.47 (1.34–1.62)	<0.001	1.35 (1.12–1.62)	0.001	1.38 (1.19–1.60)	<0.001
	Three generations	1.06 (0.93–1.21)	0.405	1.08 (0.81–1.43)	0.592	1.14 (0.91–1.42)	0.259
Tenure*Household type	Privately rented*adults with children	-	-	-	-	-	-
	Privately rented*adults with no children	1.09 (0.98–1.20)	0.105	1.03 (0.85–1.24)	0.788	1.25 (1.06–1.48)	0.009
	Privately rented*single adult with children	1.17 (0.93–1.46)	0.179	1.29 (0.82–2.04)	0.267	1.18 (0.74–1.88)	0.480
Tenure*Household type (continued)	Privately rented*single adult	1.57 (1.34–1.82)	<0.001	1.39 (1.08–1.79)	0.011	1.85 (1.46–2.35)	<0.001
	Privately rented*older cohabiting adults	1.24 (1.00–1.53)	0.052	1.37 (0.97–1.95)	0.076	1.72 (1.28–2.32)	<0.001
	Privately rented*three generations	0.96 (0.75–1.23)	0.774	0.91 (0.56–1.49)	0.720	1.15 (0.77–1.72)	0.494
	Social housing*adults with children	-	-	-	-	-	-
	Social housing*adults with no children	0.90 (0.82–0.99)	0.029	0.88 (0.74–1.04)	0.132	1.11 (0.96–1.28)	0.170
	Social housing*single adult with children	1.22 (0.98–1.52)	0.079	1.38 (0.90–2.14)	0.142	1.49 (0.97–2.30)	0.070
	Social housing*single adult	1.31 (1.15–1.50)	<0.001	1.07 (0.86–1.33)	0.557	1.48 (1.21–1.81)	<0.001
	Social housing*older cohabiting adults	0.76 (0.65–0.89)	<.001	0.88 (0.68–1.13)	0.315	0.87 (0.69–1.10)	0.244
	Social housing*three generations	0.91 (0.70–1.17)	0.451	0.79 (0.49–1.23)	0.318	0.93 (0.61–1.41)	0.728
